# Is a Clinical Target Volume (CTV) Necessary in the Treatment of Lung Cancer in the Modern Era Combining 4-D Imaging and Image-guided Radiotherapy (IGRT)?

**DOI:** 10.7759/cureus.466

**Published:** 2016-01-23

**Authors:** Jeremy M Kilburn, John T Lucas, Michael H Soike, Diandra N Ayala-Peacock, Arthur W Blackstock, William H Hinson, Michael T Munley, William J Petty, James J Urbanic

**Affiliations:** 1 Radiation Oncology, Wake Forest School of Medicine; 2 Medicine, Division of Hematology/Oncology, Wake Forest School of Medicine; 3 Radiation Medicine and Applied Sciences, UCSD School of Medicine

**Keywords:** lung cancer, radiation treatment planning, non-small cell lung cancers (nsclc), clinical target volume, image-guided radiation therapy

## Abstract

Objective: We hypothesized that omission of clinical target volumes (CTV) in lung cancer radiotherapy would not compromise control by determining retrospectively if the addition of a CTV would encompass the site of failure.

Methods: Stage II-III patients were treated from 2009-2012 with daily cone-beam imaging and a 5 mm planning target volume (PTV) without a CTV. PTVs were expanded 1 cm and termed CTVretro. Recurrences were scored as 1) within the PTV, 2) within CTVretro, or 3) outside the PTV. Locoregional control (LRC), distant control (DC), progression-free survival (PFS), and overall survival (OS) were estimated.

Result: Among 110 patients, Stage IIIA 57%, IIIB 32%, IIA 4%, and IIB 7%. Eighty-six percent of Stage III patients received chemotherapy. Median dose was 70 Gy (45-74 Gy) and fraction size ranged from 1.5-2.7 Gy. Median follow-up was 12 months, median OS was 22 months (95% CI 19-30 months), and LRC at two years was 69%. Fourteen local and eight regional events were scored with two CTVretro failures equating to a two-year CTV failure-free survival of 98%.

Conclusion: Omission of a 1 cm CTV expansion appears feasible based on only two events among 110 patients and should be considered in radiation planning.

## Introduction

The original dose-finding RTOG study for non-small-cell lung cancer used traditional two-dimensional planning with standard field arrangements based on the location of the primary [1]. Treatment delivery and, specifically, target delineation have evolved dramatically since this time with the recent developments in image-guided radiotherapy (IGRT), treatment planning based on 18F-fluorodeoxyglucose-positron emission tomography/computed tomography (PET/CT), and individualized tumor motion with four-dimensional (4-D) CT [2-5]. The ICRU Report 62 (1999) reflects some of these changes with the creation of the internal target volume (ITV), accompanying the traditional clinical target volume (CTV) and planning target volume (PTV).

A CTV expansion is generally a volumetric expansion. Pathologic data suggests microscopic disease, and hence, the CTV rarely falls outside of 1 cm from the radiographically defined gross tumor volume (GTV) [6-10]. This concept predates the majority of PET/CT, 4D imaging, and IGRT technological advances, and thus, controversy persists over target delineation and application of CTV expansions to a 4D CT-defined ITV. The variable implementation of a CTV within the cooperative groups is evidence of this controversy. The Phase II dose escalation trials, RTOG 0117 and NCCTG 0028, did not employ a CTV while CALGB 30407 and the randomized Phase III RTOG 0617 did employ a CTV [11-14]. For small cell lung cancer, the current intergroup trial (CALGB 30610/RTOG 0538) uses a hybrid approach that does not include a CTV expansion of the ITV but does include treatment of the ipsilateral hilum (ClinicalTrials.gov identifier: NCT00632853).

In order to test the hypothesis that omission of a CTV does not compromise tumor control, we evaluated outcomes from a large cohort of patients uniformly treated with 5 mm PTV expansions on the ITV to determine if the addition of a CTV would have encompassed the site of failure and potentially prevented subsequent relapse.

## Materials and methods

### Patients

Image-guided radiotherapy (IGRT) was implemented at our institution in February 2009. Through September 2012, a total of 110 Stage II-III (AJCC, Version 7) non-small cell (NSCLC) and small cell (SCLC) patients were treated with fractionated external beam radiotherapy (EBRT). Patients with Stages IA or B, node-negative Stage II (T2bN0 and T3N0), or Stage IV disease were excluded. Patient and treatment characteristics were gathered retrospectively from the electronic medical record with Wake Forest Baptist Health Institutional Review Board approval (#00022037). Patient consent was waived due to this being a retrospective chart review of patients already treated and consented at the time of treatment.

### Treatment

Patients were treated with 3D conformal (3DCRT) or intensity-modulated radiotherapy (IMRT). The GTV was defined as all abnormal appearing tumor tissue in the lung and mediastinal lymph nodes. Lymph nodes were generally included if either hypermetabolic on PET/CT or larger than 1 cm on the short-axis. PET/CT imaging for target delineation via image registration and fusion with the CT simulation was used in 95% of patients. Four-dimensional CT scans were completed on all patients to define the ITV expansion of the GTV. The envelope of gross tumor motion was delineated by using a maximal intensity projection of the 4D CT and then modifying these contours by visual verification of the coverage on each phase of the 4D scan. No CTV expansion to the ITV was applied. A 5 mm PTV expansion was then applied.

Generally, a prescription goal of 95-100% of the treatment dose was prescribed volumetrically to the PTV with heterogeneous dose calculations. Adherence to dose constraints was consistent with most published guidelines [15-16]. Patients were treated with 4 to 6 beams using 3D conformal principles and 6 or 10 MV beam energies. Small cell lung cancer patients were treated with 45 Gy at 1.5 Gy per fraction twice daily (n=5, 30%) or between 60-70 Gy in 1.8 to 2 Gy fractions once daily (n=12, 70%). Treatment greater than 2 Gy per fraction was implemented for hilar nodal disease if chemotherapy was not to be administered based on the discretion of the treating radiation oncologist. Daily image guidance was accomplished with cone-beam CT (CBCT). Gating or active breathing control was not used. The majority of patients were under the care of a single radiation oncologist (95%). 

### Follow-up

All patients were evaluated clinically and underwent a chest CT at six to eight weeks following treatment. Following this initial evaluation, patients were seen generally every three to four months for the first two years and at six-month intervals thereafter. Radiographic and clinical information was systematically reviewed to score the initial and all subsequent failures as local, regional, or distant. Radiographic response was evaluated according to the Response Evaluation Criteria in Solid Tumors (RECIST), v1.1. PET/CT scans or biopsy was employed to assist with differentiating radiation-related lung changes with recurrence. Date of treatment failure was scored as either the date of the initial scan documenting growth or increased hypermetabolic activity or the date of pathologic evaluation. Toxicity was graded retrospectively according to Common Terminology Criteria for Adverse Effects (CTCAE), version 4.0.

### Analysis of CTV failure

**Figure 1:**
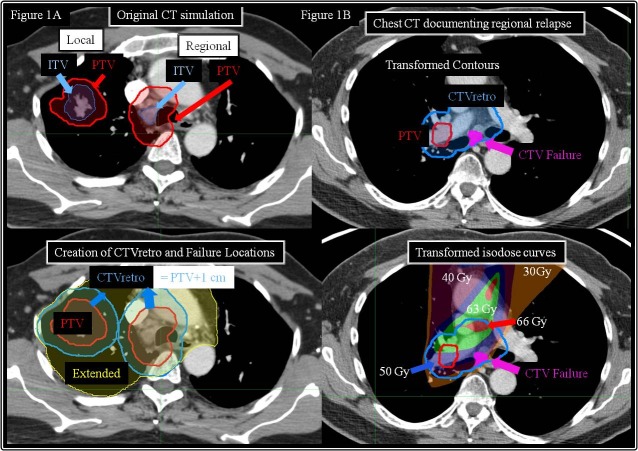
Schematic of CTV creation for analysis 1A) Creation of CTVretro for documentation of failures as PTV, CTV, or extended. 1B) Patient with documented CTV failure.

Each local and regional failure was individually reviewed by two physicians, including the principal investigator. Using MIM® Software, v5.6 (MIM Software Inc, Cleveland, OH), the simulation CT was co-registered and fused with the PET/CT or chest CT where relapse was documented for each local and regional failure. A 1 cm uniform expansion from the PTV was then retrospectively applied and termed CTVretro. Normal anatomical contours, target volumes (including the CTVretro), and isodose curves were overlaid onto the new CT. For each local and regional relapse, isodose curves, the original PTV contour, and the CTVretro contour were compared against the location of relapse and scored in one of three categories: 1) within the PTV (PTV failure), 2) outside the PTV but within the CTVretro (CTV failure), or 3) more than 1 cm from the PTV (extended failure). A patient was deemed to have a CTV failure if the CTVretro expansion would have encompassed the site of relapse, which was hypothesized to represent the scenario where a CTV could have theoretically prevented relapse. An illustration of how the CTVretro was created and of a patient experiencing a CTV failure is depicted in Figure 1.

### Statistical analysis

Local and regional control (LRC), distant control (DC), progression-free survival (PFS), and overall survival (OS) were estimated with the Kaplan-Meier method. Freedom from PTV, CTV, or extended failure was similarly derived. The completion date of EBRT was used as time zero. Patients were assessed for patient (age, sex, race), disease (stage grouping, T and N stage, histology), and treatment-related factors (dose, fraction size, PET/CT based planning, chemotherapy use, agent, and schedule) impacting the rate of local-regional failure-free survival (LRFFS) with strata comparisons using the log-rank statistic.

A univariate analysis (UVA) evaluated the association of patient, disease, and treatment-related covariates on LRFFS after assessing for proportional hazards assumptions. Clinically relevant and statistically significant covariates at the p=0.2 level were included in a multivariate analysis (MVA). All statistical measures were performed in SAS software, v9.2 (SAS Institute, Cary, NC).

## Results

### Patients

Patient characteristics are outlined in Table 1. A total of 110 lung cancer patients were reviewed. Median follow-up was 12 months (2-44 months). Median age was 65 years. Distribution across stages was IIA in 4%, IIB in 7%, while Stage IIIA and IIIB made up a further 57% and 32%, respectively. Adenocarcinoma and squamous cell carcinoma were equally represented and comprised the majority (70%) of all patients.

**Table 1:** Patient Characteristics N=Number, %=Percent

			N (%)
Patient Characteristics			N=110
Age			
Median			69
Range			38-87
>70 Years			55 (33)
ECOG			
0-1			78 (71)
2			24 (22)
3			8 (7)
Race			
Caucasian			90 (82)
African-American			20 (18)
Sex			
Male			103 (64)
Female			59 (36)
Group Stage			
IIA			4 (4)
IIB			8 (7)
IIIA			63 (57)
IIIB			35 (32)
T Stage			
1			27 (25)
2			38 (35)
3			16 (15)
4			25 (23)
x			4 (4)
N Stage			
0			12 (11)
1			15 (14)
2			58 (53)
3			25 (23)
Histology			
Adenocarcinoma			38 (35)
Squamous Cell			39 (35)
Large Cell/Neuroendocrine			3 (3)
NSCLC NOS			10 (9)
Small Cell Lung Cancer (SCLC)			17 (15)
Other/Unknown			3 (3)

### Treatment

Treatment characteristics are detailed in Table 2. The median radiation dose was 70 Gy and 2.0 Gy per fraction (fx). Intensity-modulated radiotherapy was used in 14 patients (13%). Accelerated hypofractionation (AHFX), defined as greater than 2.0 Gy/fx, was delivered in 14%. Of those treated with AHFX, only four of these patients were treated in the mediastinum for N2 nodal disease with the remaining patients treated for hilar nodal disease. Chemotherapy was delivered to 78% of patients, concurrently to 86% of Stage III patients.

**Table 2:** Treatment Characteristics N = Number, % = Percent, Fx = Fraction a: Values and percentages exclude patients treated with 45 Gy BID for SCLC (N=5). b: Only 4 of the 15 were treated to the mediastinum with N2 disease. c: Values and percentages limited to Stage II patients. d: Values and percentages limited to Stage IIIA/B patients. e: Percentage values represent percent of those receiving chemotherapy.

			N (%)
Treatment Characteristics			N=110
PET Planned			104 (95)
Radiation Dose			
Median			70 Gy
Range			45-74 Gy
> 60 Gy^a^			104 (99)
> 70 Gy^a^			56 (53)
Fraction Size			
Median (Range)			2 Gy (1.5-2.7 Gy/Fx)
>2.0 Gy/Fx			15 (14)^b^
Chemotherapy (Any)			86 (78)
Stage II^c^			2 (17)
Stage III			84 (86)
Induction^d^			17 (17)
Concurrent^d^			76 (78)
Adjuvant^d^			13 (13)
Chemotherapy Agent			
Carboplatin and Paclitaxel^e^			51 (59)
Carboplatin/Cisplatin and Etoposide^e^			17 (20)
Carboplatin and Pemetrexed^e^			10 (12)
Other/Unknown^e^			8 (9)

### Patterns of failure

Local-regional control (LRC) at one and two years was 85% and 68%, respectively. Local failure-free survival (LFFS) and regional failure-free survival (RFFS) at two years were 75% and 88%, respectively. Among patient and disease characteristics using UVA, advancing age (HR 1.05, 95% CI 1.00-1.09) was associated with an increased hazard for local-regional failure (LRF) while a trend was noted with squamous cell carcinoma histology (HR 2.05, p=0.12). Group stage did not impact the hazard for LRF (HR 0.78, p=0.45) nor did advancing T and N stages (p=0.39 and p=0.78). Several treatment-related variables were predictive, including the use of any chemotherapy and concurrent delivery with hazard ratios for LRF of 0.34 (95% CI 0.13-0.89) and 0.36 (95% CI 0.15-0.90), respectively. Although total radiation dose was not predictive, use of AHFX increased the hazard for LRF (HR 3.00, 95% CI 1.06-8.52). When age, stage, squamous histology, treatment with AHFX, and use of chemotherapy were included in the construction of an MVA, only age retained statistical significance with advancing age predictive for increased hazard for LRF (aHR 1.09, 95% CI 1.02-1.16).

### Analysis of CTV failures

Scoring local and regional failures as separate events, a total of 22 total failures were observed. Isolated local failure (LF) and isolated regional failure (RF) occurred in 12 and six patients, respectively. Only two patients recurred with both an LF and simultaneous RF (LRF). Among the 14 patients with LF, 12 (86%) were scored as PTV failures, while the remaining two patients failed within the same lobe of the lung but greater than 1 cm from the PTV and were thus scored as an extended failure. Extended failures made up four of the eight regional failures (50%), with two patients failing each in the PTV and CTVretro volumes (Table 3).

**Table 3:** Treatment Outcome and Patterns of Failure N=Number, %=Percent a: Percentage value represents percent of local or regional failure. b: Percentage value represents percent of failures.

		Cumulative Incidence	First Site of Failure	2 Year Failure Free Survival
		N=110 (%)	N=110 (%)	N=110 (% (95% CI))
Any Failure		57 (52)	57 (52)	32% (21-43)
Local		14 (13)	13 (23)^b^	75% (59-85)
	PTV failure^a^	12 (86)		77% (62-87)
	CTV failure^a^	0		100%
	Extended failure^a^	2 (14)		98% (90-99)
Regional		8 (7)	4 (7)^b^	88% (77-94)
	PTV Failure^a^	2 (25)		97% (86-99)
	CTV Failure^a^	2 (25)		98% (92-99)
	Extended failure^a^	4 (50)		94% (83-98)
Locoregional		20 (18)		68% (53-79)
Distant		43 (39)	39 (68)^b^	51% (38-63)
Simultaneous Sites			1 (2)^b^	
Overall Survival				48% (36-59)

**Figure 2:**
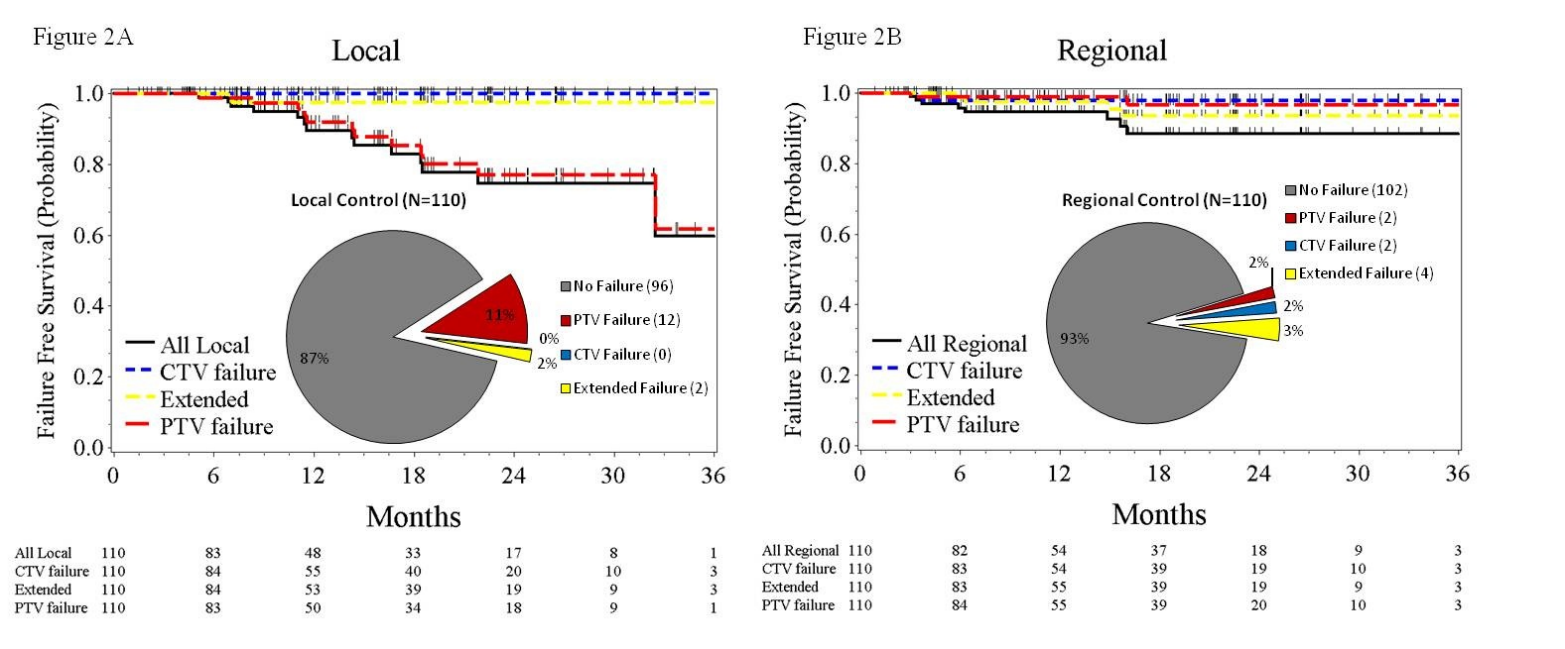
Local and Regional Failures 2A) Kaplan-Meier plot and pie chart documenting failures and failure-free survival from any local failure, PTV failure, CTV failure, or extended local failure. 2A) Any regional failure, PTV failure, CTV failure, or extended regional failure.

Only two of the 22 failures, both with RF, were determined to have failed in the retrospectively-derived CTV expansion and were thus deemed a CTV failure. The chest CT used to document failure in one of the two patients is illustrated in Figure 1. The corresponding rates of freedom from local or regional CTV failure were 100% and 98%, respectively (Figure 2).    

### Toxicity

Toxicity gathered retrospectively from the clinical return visits was as follows: any grade acute or late (> 90 days post treatment) toxicity was seen in 85% of locally advanced patients, with acute and late toxicities noted in 82% and 30%, respectively. Among acute toxicities, the vast majority was Grade 1-2, as high-grade toxicity (Grade > 3) was seen in only 9%. No acute treatment-related deaths occurred. Esophagitis was the most common acute toxicity and accounted for all high-grade toxicity. Other relevant toxicities included any grade fatigue and dehydration in 21% and dyspnea in 9%. Regarding late toxicity, high-grade toxicity was seen in only 6%. Esophageal stricture and lung fibrosis made up an equal proportion of these patients. A single patient died of late treatment-related lung fibrosis/respiratory failure.    

### Survival analysis

**Figure 3:**
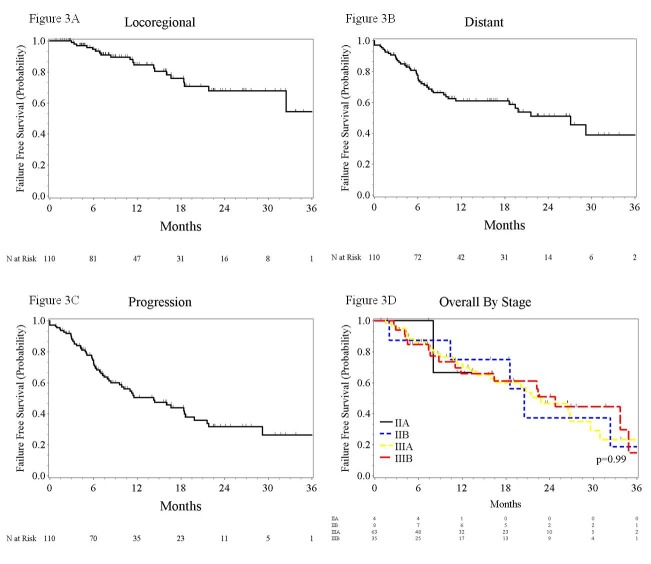
Survival Curves 2A) Kaplan-Meier plots for locoregional, 2B) distant failure-free survival, 2C) progression-free survival, and 2D) overall survival by stage.

Median overall survival (OS) was 22 months (95% CI 18.6-29.6 months) with one and two-year OS rates of 70% and 48%. Advancing stage was not associated with diminished survival rates (p=0.85). Adenocarcinoma histology was associated with an increased hazard for death (HR 1.98, 95% CI 1.11-3.51). Dose (p=0.25) and fraction size (p=0.93) did not alter the hazard for death while the use of concurrent chemotherapy was predictive (HR 0.51, 95% CI .28-.90). Median progression-free survival (PFS) was 14 months (95% CI 8.4-18.6 months). Distant failure-free survival (DFFS) at one and two years was 61% and 51%, respectively. Neither PFS (p=0.85) nor DFFS (p=0.27) rates differed between group stages. Figure 3 depicts survival plots of local/regional failure-free survival (LRFFS), PFS, DFFS, and OS by stage.

## Discussion

Our analysis revealed a crude 2% failure rate within 1 cm from the PTV correlating to an estimated 99% failure-free survival probability at two years. Only two among a cohort of 110 patients or two of the 22 failure events were scored as being potentially due to lack of a CTV. Although local and regional events were noted in a significant amount of patients, the location of these failures suggests the omission of a CTV did not sacrifice locoregional control.  

The fundamental concept of CTV is coverage of microscopic tumor extent. Pathologic series have shown conventional CT to define this region with only a fair degree of accuracy, yet in some cases is overestimated [6]. Other groups have found a high correlation between imaging and pathologically-defined tumor extent [8-9]. This correlation has been even further defined when incorporating PET/CT imaging [7, 10]. These data, unfortunately, fail to account for respiratory motion and, therefore, may not be fully applicable in the era of 4D planning and image-guided radiotherapy. However, those datasets provide the useful information that the pathologic extent of tumor rarely exceeds 1 cm from the radiographic findings. 

In their pathologic series, Grills, et al. appropriately highlighted another important point emphasizing the effect of the penumbra on the dose to targets where the 80% isodose line covers up to 8.5 mm outside of the PTV [6]. Their series was limited to SBRT treatments where the dose cloud was more conformal compared to the typical four to six beams used in conventional EBRT and, therefore, underestimated the integral dose to disease below the detection limits of modern imaging in close proximity to the PTV. The lack of failures within 1 cm of the planning volume in our study underscores the possible impact of penumbra coverage of subclinical microscopic disease.

All patients in our analysis were treated in similar fashion as discussed in the Methods section of this report with 4D CT imaging and daily IGRT with CBCT used in all patients with PET/CT planning in the vast majority. Furthermore, contouring variations were minimized with a single radiation oncologist responsible for 95% of treatments. The homogeneity among treatments is both an advantage and disadvantage when considering the applicability of our conclusions. It is possible that a potential contouring bias occurs with a single physician being responsible for these patients, which cannot be well controlled for. In other words, advanced radiotherapy planning likely contributed substantially to our outcomes and whether the omission of a CTV is feasible without these technologies or whether our results can be broadly generalized if others with similar technology adopted this approach is unknown. This is potentially a good example of the clinical scenario in which continual reassessment of a provider’s outcome and quality data would be valuable.

Although relapse within 1 cm of the PTV was rare, variations were seen between relapses within the PTV or more than 1 cm from the PTV (extended failure). The majority (86%) of patients failing locally relapsed within the PTV contour while half of regional relapses were experienced more than 1 cm from the PTV. This information suggests failures at the primary site are a consequence of ineffective tumor cell kill and not a result of inappropriate target delineation. Unfortunately, local failures are a significant issue in this patient population. As corroborated by our findings on UVA, multiple prospective trials and a meta-analysis demonstrated improved survival with concurrent chemotherapy based largely on improved locoregional control [17-18].

Dose-escalation strategies for Stage 3 disease have proven to be tricky. While SBRT has improved control rates in early stage cancers, this technology is not appropriate for central tumors or locally advanced disease [19-22]. Recent retrospective and prospective Phase II publications aimed at improving locoregional control in locally advanced disease have demonstrated the feasibility of dose escalation. However, the high-dose arm in the randomized Phase III comparison of 60 Gy versus 74 Gy (RTOG 0617) was prematurely closed when futility boundaries were crossed [14, 23-27], and now more mature results have shown 74 Gy to be associated with decreased local control and survival [28].

Regarding regional targets, any relapse was uncommon with a two-year RFFS rate of 88%. This suggests both sufficient tumor cell kill and successful targeting. The rare regional failure further substantiates the omission not only of a CTV but of elective nodal irradiation (ENI). Both prospective and retrospective studies have demonstrated the safety of ENI omission and led to its elimination from most current national protocols [29-30]. While ENI has largely been abandoned, CTV expansions are commonly implemented in recent and ongoing trials, such as RTOG 0617, where 0.5 to 1.0 cm expansions from either the GTV or ITV were applied [14].    

The successful omission of a CTV is predicated on two assumptions created from our reliance on both technology and the human element: 1) sufficient sensitivity of diagnostic imaging modalities to identify disease and the technology to precisely target this region over a full radiation course, and 2) the radiation oncologist accurately identifying disease sites on the simulation CT scan and ensuring adequate dosimetric coverage with the assistance of the dosimetrist. Substantial evidence has lent support for PET/CT planning with significant alterations noted in planning volumes compared to CT-based plans alone [31-33]. Furthermore, 4D CT and IGRT have diminished inter and intra-fraction uncertainty by making smaller margins feasible and safe [2-5]. The combination of these technologies was applied in nearly all patients in our analysis. Ensuring the disease identified is appropriately contoured is another obstacle and potential argument for the application of a CTV. Fittingly, significant intra and inter-clinician variations in target volumes have been demonstrated as well as variations between targets outlined by radiologists and radiation oncologists [34-35]. Therefore, an acknowledged weakness of our analysis is the inability to quantify if the contouring technique of the treating radiation oncologist is fully generalizable or if the lack of failures attributable to the CTV omission can be attributed to their individual technique and experience. This point was illustrated by our own intra-clinician evaluation of one of the two CTV failures, which was retrospectively deemed to be inadequately contoured. Once again, however, although variations in contours exist, inadequately contoured structures are even less frequent than CTV failures, as demonstrated by our findings and, therefore, do not support the use of CTV expansions to avoid contouring errors.

Although the primary goal of lung cancer treatment is preventing relapse and ultimately improving survival, minimizing toxicity is also an important endpoint. The survival benefit of concurrent chemotherapy came at the cost of increased toxicity, mainly Grade 3 esophageal injury seen in approximately 20% [17-18]. While ENI was used in the chemotherapy trials, the subsequent omission of ENI provided smaller irradiated volumes intended to minimize toxicity while maintaining similar or better control rates. We are now seeing a similar trend through the omission of a CTV. The omission of a 1 cm expansion underscores the impact on the irradiated volume, considering the influence on the volume of the spherical expansion [4/3πr^3^]. The 1 cm expansion illustrated for the patient in Figure 1 increased the target volume from 226.8 cc to 694.1 cc. Unfortunately, the improved therapeutic ratio is theoretical as a comparative toxicity analysis between patients treated with and without a CTV is not possible. Although the retrospective nature of our analysis hinders definitive conclusions, our low 9% acute high-grade toxicity rate helps substantiate this theoretical advantage.

## Conclusions

The addition of a 1 cm CTV expansion would infrequently prevent recurrence based on only two events among 110 patients in our analysis. For patients treated with the modern advances of 4D CT, IGRT, and PET/CT planning, the omission of larger expansions is feasible. The therapeutic ratio is theoretically improved by the omission of a CTV and should be considered in the design of future clinical trials.
